# Supplementary material for: ProteinShader: illustrative rendering of macromolecules
Source: BMC Struct Biol. 2009 Mar 30;9:19. doi: 10.1186/1472-6807-9-19 (PMC2672931; doi:10.1186/1472-6807-9-19)
Supplement: Additional file 1 — ProteinShader program without source code. This compressed file contains the complete ProteinShader program including associated libraries, but no source code. A README.txt file gives an overview of the ProteinShader distribution, and the index.html file in the help subdirectory has directions on getting started with the program as well as a set of tutorials. [file 1472-6807-9-19-S1.zip › ProteinShader-beta-0_9_4-binary/help/api/overview-summary.html]

Overview (ProteinShader API)


|  |  |  |
| --- | --- | --- |
| **Overview** \| Package \| Class \| Use \| **Tree** \| **Deprecated** \| **Index** \| **Help** | |  |
| PREV   NEXT | **FRAMES**    **NO FRAMES**     **All Classes** |  |


---


# ProteinShader Beta 0.9.4 API Specification

The ProteinShader program is a free open-source platform-independent molecular
visualization tool designed to produce illustrative renderings of proteins
that approximate what an artist might create using pen and ink.

**See:**
  
          **Description**

| **Packages** | |
| --- | --- |
| **org.proteinshader.graphics** | Holds the drawing classes: Ribbon, Tube, FrenetFrames, Sphere, and Cylinder. |
| **org.proteinshader.graphics.adapter** | Holds the StructureToGraphics class, which is used to manage the use of the drawing classes and OpenGL display lists. |
| **org.proteinshader.graphics.displaylists** | Holds the classes needed to manage OpenGL display lists, which are used to cache reusable geometry for spheres, cylinders, ribbon segments, and tube segments. |
| **org.proteinshader.graphics.enums** | Holds the enums needed by org.proteinshader.graphics and its subpackages. |
| **org.proteinshader.graphics.exceptions** | Holds the exception classes needed by org.proteinshader.graphics and its subpackages. |
| **org.proteinshader.graphics.shader** | Holds the classes needed for compiling and managing the vertex and fragment shaders used for texture mapping and custom lighting calculations. |
| **org.proteinshader.graphics.textures** | Holds the classes needed for creating and managing OpenGL texture objects. |
| **org.proteinshader.graphics.typography** | Holds the classes needed for applying text labels to the curved surfaces of tubes and ribbons. |
| **org.proteinshader.graphics.utils** | Holds a testing utility, class SegmentRenderer, which can be used for drawing ribbon and tube segments on-the-fly, rather than using cached geometry in the form of OpenGL display lists. |
| **org.proteinshader.gui** | Holds all of the Swing GUI components and their associated listeners, including class Renderer, which is registered as a listener for the GLCanvas object that is used a drawing surface. |
| **org.proteinshader.gui.components** | Holds the gui components (JPanels and JMenus) needed by class ProteinShaderGUI. |
| **org.proteinshader.gui.components.controlpanel** | Holds the JPanels used in the right-side retractable control panel of the GUI. |
| **org.proteinshader.gui.components.menubar** | Holds the JMenus used in menu bar above the canvas. |
| **org.proteinshader.gui.enums** | Holds the enums needed by org.proteinshader.gui and its subpackages. |
| **org.proteinshader.gui.exceptions** | Holds the exception classes needed by org.proteinshader.gui and its subpackages. |
| **org.proteinshader.gui.listeners** | Holds the listener classes for the gui. |
| **org.proteinshader.gui.listeners.controlpanel** | Holds the listeners for components used in the JPanels of the right-side retractable control panel of the GUI. |
| **org.proteinshader.gui.listeners.menubar** | Holds the listeners for the items used in the JMenus of the menu bar above the canvas. |
| **org.proteinshader.gui.utils** | Holds minor utility classes needed by the Renderer. |
| **org.proteinshader.gui.viewing** | Holds important helper classes that the Renderer uses to control the view: Camera, Rotation, Lighting, *etc*. |
| **org.proteinshader.math** | The key classes in this package are Hermite and Quaternion, which are needed for generating the ribbons and tubes that are used to represent the backbone of a protein in a cartoon-type display. |
| **org.proteinshader.structure** | Holds the classes that store information from a Protein Data Bank file: Structure, Model, Chain, AminoAcid, Heterogen, Water, Atom, Bond, Helix, BetaStrand, Loop, *etc*. |
| **org.proteinshader.structure.enums** | Holds the enums needed by org.proteinshader.structure and its subpackages. |
| **org.proteinshader.structure.exceptions** | Holds the exception classes needed by org.proteinshader.structure and its subpackages. |
| **org.proteinshader.structure.factory** | Holds the factory classes needed for generating the Segment objects that hold the information needed for rendering ribbon and tube segments. |
| **org.proteinshader.structure.io** | Holds the classes needed for reading Protein Data Bank structure files. |
| **org.proteinshader.structure.io.demos** | Holds demo classes that were used for early testing of the io classes. |
| **org.proteinshader.structure.io.enums** | Holds the enums needed for parsing Protein Data Bank structure files. |
| **org.proteinshader.structure.io.exceptions** | Holds the exception classes that may be needed when parsing Protein Data Bank structure files. |
| **org.proteinshader.structure.io.filters** | Holds filters for checking file extensions. |
| **org.proteinshader.structure.sort** | Holds the DrawableSorter class, which is needed for sorting Drawable objects based on their distance from the camera. |
| **org.proteinshader.structure.visitor** | Holds Visitor classes that know how to traverse the hierarchy of objects held by class Structure. |
| **org.proteinshader.structure.visitor.demos** | Holds demo classes that were used for early testing of the Visitor classes. |
| **org.proteinshader.structure.visitor.enums** | Holds enums needed by the Visitor classes. |
| **org.proteinshader.structure.visitor.exceptions** | Holds exception classes needed by the Visitor classes. |
| **org.proteinshader.structure.visitor.modifiers** | Holds helper classes for the ModifierVisitor class, which is used to modify Drawable objects (Atoms, Bonds, and Segments). |

The ProteinShader program is a free open-source platform-independent molecular
visualization tool designed to produce illustrative renderings of proteins
that approximate what an artist might create using pen and ink.
